# Sentence Comprehension and L2 Exposure Effects in 6‐Year‐Old Sequentially Bilingual Children With Typical Development and Developmental Language Disorder

**DOI:** 10.1111/1460-6984.70125

**Published:** 2025-09-28

**Authors:** Sini Smolander, Marja Laasonen, Pekka Lahti‐Nuuttila, Eva Arkkila, Elin Thordardottir, Sari Kunnari

**Affiliations:** ^1^ Department of Logopedics, School of Humanities, Philosophical Faculty University of Eastern Finland Joensuu Finland; ^2^ Research Unit of Logopedics University of Oulu Oulu Finland; ^3^ Department of Otorhinolaryngology and Phoniatrics, Head and Neck Surgery Helsinki University Hospital and University of Helsinki Helsinki Finland; ^4^ Department of Psychology and Logopedics, Faculty of Medicine University of Helsinki Helsinki Finland; ^5^ School of Communication Sciences and Disorders, Faculty of Medicine and Health Sciences McGill University Montreal Canada

**Keywords:** child bilingualism, classification accuracy, developmental language disorder, language assessment, language exposure, sentence comprehension, sequentially bilingual

## Abstract

**Background:**

Differentiating typical language development (TD) and developmental language disorder (DLD) in a bilingual context is difficult. The societal language is often the only mutual language of the child and the SLT. It has been shown that when assessing second language (L2) performance using tools developed for monolingual children, substantial differences between typical and disordered development can be found. There is a need for a systematic understanding of the applicability of tests across different language domains, taking language exposure into account. Sentence comprehension is an important part of language development, and receptive difficulties have often been considered to have prognostic value. Yet, the diagnostic value of sentence comprehension has received little research attention.

**Aim:**

In the Helsinki longitudinal SLI study (HelSLI), we investigated L2 exposure effects on sentence comprehension in sequentially bilingual typically developing children (BiTD) and bilingual children with DLD (BiDLD). In addition to group‐level comparisons, we examined the classification accuracy of two L2 sentence comprehension tests, taking several explanatory factors into account.

**Methods and Procedures:**

A total of 100 six‐year‐old children were recruited from day care centres and a hospital clinic (54 BiTD children and 46 bilingual children with DLD). Two offline tests with multiple‐choice and act‐out tasks (Sentence Comprehension Test (Lausetesti) and Reynell Developmental Language Scales III, Verbal Comprehension Scale) were used to investigate sentence‐level comprehension in Finnish. Multiple regression analysis was used to compare BiTD and BiDLD group performance while considering the effects of relative lifetime exposure to L2. Covariate‐specific receiver operating characteristic (ROC) analysis was used to study the classification accuracy of the two tests and estimate the thresholds for optimal sensitivity and specificity of the tests.

**Outcomes and Results:**

The TD bilingual children performed significantly better than their peers with DLD in both sentence comprehension tests. The effect of L2 exposure was significant but small and affected both groups similarly. Both tests classified the groups with fair sensitivity and specificity at their best, but the accuracy depended greatly on exposure. Depending on the age and exposure to L2, a sensitivity of 0.80 yielded a specificity of 0.16–0.87 on the Sentence Comprehension Test and a specificity of 0.19–0.87 on the RDLS III Verbal Comprehension Scale.

**Conclusions and Implications:**

Sentence comprehension in L2 is promising in informing the detection of language difficulties in L2 Finnish learners with several first‐language backgrounds. However, interpretation must take L2 exposure into account. In addition, no one assessment tool or domain can be considered enough for the identification of DLD. In the future, the importance of sentence comprehension tests as classifiers should be considered as part of a larger assessment battery.

**WHAT THIS PAPER ADDS:**

*What is already known on this subject*

- Differentiating developmental language disorder (DLD) from typical language development (TD) is difficult in bilingual children. Assessment tools are needed for more reliable detection of disordered development. It has been shown that assessing both languages in bilingual children adds to the classification accuracy. The societal language is, however, often the only mutual language between the SLT and the child, and the only available tests are frequently in the child's L2. It has been found that substantial differences in second language (L2) performance between bilingual typically developing children (BiTD) and bilingual children with DLD (BiDLD) groups can be found in different language domains. Language exposure effects in BiTD and BiDLD children as well as classification accuracy of the L2 tests vary, however.

*What this paper adds to existing knowledge*

- L2 sentence comprehension has received little attention in the field of communication disorders. In the current study, sentence comprehension tests showed substantial differences in L2 performance between bilingual TD and DLD children. On a group level, the effect of L2 exposure was small relative to some other language domains and appeared similar in BiTD and BiDLD children. Classification accuracy, however, depended greatly on exposure. In addition, it was found that some tests classify the children with more exposure better and some those with less exposure. Such patterns are related to the difficulty of the test items and the age range targeted by each test.

*What are the potential or actual clinical implications of this work?*

- Sentence comprehension is a promising language domain informing the identification of DLD in bilingual children with different L1 backgrounds. Even though the BiTD and BiDLD groups differ significantly in their test performance, it is advisable to consider the possible differences in sensitivity and specificity of the test depending on exposure. In the domain of sentence comprehension, despite the persistent difference in performance between BiTD and BiDLD children, DLD children seem to be able to benefit from the exposure in a similar manner as TD children instead of lagging behind in the course of accumulating exposure.

## 1 Introduction

Differentiating typical language development (TD) from developmental language disorder (DLD) in varying bilingual settings has been found to be very challenging due to limited diagnostic instruments. In the absence of standardised tests for every bilingual setting and language pair, clinical professionals often use tests developed for monolingual children to study second language (L2) performance. Even when these tests are used without referring to the monolingual norms and are instead considered qualitatively as a complement to other assessment tools and frameworks (e.g., interviews, observations, language samples, processing measures, dynamic assessment), interpreting performance based on L2 tests is restricted. This is due to the lack of reference points from typically developing bilingual children and especially ones that take L2 exposure into account (see, however, Elin Thordardottir [2015] for clinical procedures proposed for pro‐rating cut‐off criteria based on the individual child's previous exposure to the language being evaluated).

Sentence comprehension has rarely been studied in the context of bilingualism and especially in bilingual children with DLD (see, however, De Cat and Melia [2022]; Elin Thordardottir et al. [2006]; Rusk et al. [2020]; and Unsworth [2016] for comprehension in bilingual typically developing children [BiTD] and Bonifacci et al. [2020]; Gillam et al. [2013] for bilingual children with DLD [BiDLD]). This is a significant gap, considering that efficient communication and learning require sentence‐level processing, and children with DLD often exhibit limitations in cognitive and linguistic abilities that affect sentence comprehension (Montgomery et al. 2021). These oral comprehension difficulties, in turn, predict later language development, reading comprehension, and school achievement (Adlof and Catts 2015; Conti‐Ramsden et al. 2009; Montgomery et al. 2021), and in some studies, they are connected to a poorer quality of life even in adulthood (Arkkila 2009). Hence, including an evaluation of comprehension is strongly recommended in the diagnostic assessment (Bishop et al. 2017). The applicability of clinical tests tapping comprehension across different stages of L2 exposure, however, is unknown.

In this study, we investigated L2 sentence comprehension performance and second language exposure effects in sequentially BiTD and BiDLD children who have different first language backgrounds. Furthermore, we explored the applicability and restrictions of using and interpreting standardised sentence comprehension tests developed for monolingual children. In addition to informing the diagnostic procedure, this study adds to the understanding of sentence comprehension performance as a function of accumulating L2 exposure in BiTD and BiDLD groups. This, in turn, enables clinicians to adjust their expectations for language performance and base them more on a bilingual mindset (Elin Thordardottir 2015).

## 2 Sentence Comprehension in Bilingual Typical Development

An important aspect of the comprehension of a single sentence is how semantic roles are assigned; at its simplest, it is identifying who did what to whom. According to the framework of Competition and Unified Models (see MacWhinney 2005), people comprehend the meanings of sentences based on linguistic cues that are available and reliable in showing relationships between the words, linguistic forms, and functions in that particular language. For example, agglutinative languages, including the non‐Indo‐European Finno‐Ugric languages Finnish, Estonian and Hungarian, as well as the Turkic language Turkish, make extensive use of case markings as cues in sentence comprehension (Hyönä and Hujanen 1997; MacWhinney and Pléh 1988; Slobin and Bever 1982).

In these languages, a series of inflections with their own grammatical function are attached to the stem (Leonard 2014). Hence, words can typically become quite long. The order of the inflections is usually fixed, while word order is relatively free. Agglutinating languages have several cases. Finnish has about 15 cases (Karlsson 2015). Grammatical cases mark grammatical roles such as agent, patient or recipient, and semantic cases mark adverbials and have a rather concrete meaning, for example, location (Huumo et al. 2023; Kittilä et al. 2022). Morphology and syntax are heavily intertwined (Hyönä and Hujanen 1997). The word forms in all the agglutinating languages discussed in this section are considered to be easy to analyse if one knows the endings. This might have implications for L2 learners’ as well as DLD children's developing morphosyntax (see Elin Thordardottir (2016) for an overview on grammatical morphology as a marker for DLD). However, while agglutinating languages share grammatical characteristics (e.g., a rich system of noun and verb inflections), they are also in some respects different from each other (see, for example, Dasinger 1997; Leonard 2014). For example, when comparing Finnish and Hungarian, two languages from the same language family, Finnish permits null subjects only for the first and second person in the verb paradigm, unlike Hungarian. There is also a requirement for person and number agreement with the subject but no definiteness agreement with the object, like in Hungarian (Leonard 2014).

Bilingual children need to process and become proficient in two linguistic systems. In order to comprehend a second language efficiently, children need to learn to integrate morphosyntactic cues from their languages. Early L2 development has been found to promote amalgamation as the most frequent cue use pattern (Reyes and Hernández 2006). In that, cues which are moderately strong in both languages are used in combination to interpret sentences in both languages.

How much and in which ways languages are influencing each other in bilingual language development is modulated by language overlap and language dominance (Van Dijk et al. 2022). Some studies have found cross‐linguistic influence even in the case of no overlap in morphosyntactic properties, but most often, partial overlap is observed (Van Dijk et al. 2022). For example, when studying the processes of lexical morphology in Finnish L2, Vainio et al. (2014) found more efficient processing of semi‐transparent morphemes (word stems alternating according to the suffix attached to it) in Finnish comprehension for Russian L1 (first language) speakers compared to Chinese first language (L1) speakers, since the cue validity of inflectional suffixes is high in Finnish and Russian but not in Chinese. This was true although the languages in question are not related.

Whereas monolingual Finnish‐speaking children are rather quick in learning the rich morphological system of their native language (Kunnari et al. 2016), how morphosyntactic skills in L2 Finnish develop is not known. It could be hypothesised that for typically developing bilingual children, Finnish possesses some facilitative features. It has been suggested that grammatical role assignment could be less burdensome for the memory when this assignment is possible immediately when the word is recognised (see, however, Hyönä and Hujanen (1997) for a discussion about Finnish as a local cue language). In addition, the pattern of inflectional forms appearing frequently in speech input and the ease of segmenting the morphemes because they often have clear meanings and occur in word‐final position (Kunnari et al. 2016) might mitigate the effort in learning to understand Finnish morphosyntax. In addition, if we assume that performing well in sentence comprehension requires inferencing, verbal reasoning, memory and attention skills as well as visual literacy (De Cat and Melia 2022; Montgomery et al. 2021), typically developing bilingual children might perform reasonably well despite the level of L2 exposure. This study provides an initial look at bilingual children's comprehension of Finnish sentences.

## 3 Characteristics and Explanations of Sentence Comprehension in DLD

DLD, previously referred to as specific language impairment (SLI), refers to enduring difficulties in language that are not connected to any known biomedical condition (Bishop et al. 2017). It is a significant limitation in language ability exhibited by approximately 5%–7% of the population at the age of 5 years (Tomblin et al. 1997). DLD frequently affects language performance beyond childhood and has a significant effect on social interactions, education, employment and mental health (Arkkila et al. 2009; Bishop et al. 2017; Reilly et al. 2018). This is especially true for DLD with receptive difficulties (Conti‐Ramsden et al. 2009).

In addition to often low vocabulary size affecting comprehension of spoken language in children with DLD (Jones and Westermann 2021), trouble building long‐distance syntactic relationships has been described as one of the hallmarks of DLD (Montgomery et al. 2021). This means difficulties keeping track of grammatical relationships extending across many unrelated words within and between clauses (Hsu et al. 2014). From this perspective, many types of sentences have been reported to present difficulties for the spoken language comprehension of children with DLD, for example, wh‐questions, reversible sentences, certain types of passives, pronominal and reflexive sentences, and object relative sentences (see e.g., Girbau 2018; Hsu et al. 2014; van der Lely and Pinker 2014). To give an example, in the sentence ‘The cat that the dog chased fled up the tree’, a long‐distance dependency is created between the subject and the verb. Even though sentence comprehension studies in bilingual contexts are fewer compared to monolingual settings, similar difficulties in establishing long‐distance grammatical relations for sentence comprehension have been reported in BiDLD children. For example, Girbau (2018) found similar difficulties in bilingual Spanish‐English‐speaking 7–10‐year‐old children and monolingual children with DLD in the comprehension of direct object and reflexive pronoun sentences.

Language disorders with somewhat similar profiles across several languages have been reported by now. However, it has been shown that there are differences in how DLD manifests itself in different languages (Leonard 2014). Moreover, studies about environmental influences on language development and specifically on the effects of exposure in bilingual children with DLD are still scarce but might offer new insights into DLD since they might differ across languages (Ebert and Reilly 2022). Hence, describing and quantifying language abilities in different languages is vital for further development of assessment and treatment in bilingual settings.

To our knowledge, there are no systematic studies on sentence comprehension in children with DLD nor in typically developing children acquiring Finnish. However, in studies addressing the production of morphological forms in TD children learning agglutinating languages, it has been shown that there might be some advantages of a rich inflection system in learning morpho‐syntax (Elin Thordardottir 2016; Leonard 2022). In addition, there is research about challenges in producing morphological forms in DLD (Kunnari et al. 2011; Leonard 2014). This might be relevant, since in Finnish, case markings used as cues in sentence comprehension, morphology and syntax are highly intertwined, and it has been shown that children with DLD are likely to make similar errors in morphosyntactic comprehension and production (Rice et al. 1999). In their studies, Kunnari et al. (2011) and Leonard et al. (2014) found that even though typically developing Finnish children learn to produce inflections early, children with DLD face challenges in subject‐verb agreement and case agreement (suffixes marking accusative, partitive and genitive). Morpho‐phonological alternations in Finnish might also contribute to the problem documented in DLD. Morpho‐phonological alternations mean that the words may have different stems depending upon which ending is to follow (Leonard 2014).

In addition to the challenges in linguistic‐specific abilities, sentence comprehension has been suggested to be affected by difficulties in more general cognitive processing abilities, for example, different aspects of memory (see, e.g., Montgomery et al. 2021). In their structural model of sentence comprehension, Montgomery et al. posit that children with DLD process the input inefficiently because they do not have multiword templates to activate from their long‐term memory to help them chunk the message. When comprehension takes place word‐by‐word and mostly through controlled attention and working memory, it becomes effortful. Similarly, in their recent study, Hsu et al. (2014) have reported difficulties in the implicit learning of probabilistic patterns affecting the acquisition of non‐adjacent dependencies. Attributable to an underlying statistical learning deficit, Jones and Westermann (2021) adduce a predictive processing hypothesis as one addition to the explanations in which they posit a difficulty in the ability to anticipate upcoming syntactic features. All these aforementioned cognitive challenges apply to bilingual and monolingual children with DLD. Hence, sentence comprehension measured by offline picture selection tasks, which involve a number of different processes, might be informative in differentiating TD and DLD in bilingual children. The effects of factors specific to bilingual development and possibly affecting sentence comprehension, namely exposure to L2, are not known.

We expect bilingual children to exhibit similar patterns in acquiring skills in L2 comprehension to those seen in monolingual Finnish‐speaking children in morpho‐syntactic production, namely, fluent acquisition of forms and structures in the BiTD group and challenges in the BiDLD group. However, comparisons to monolingual children or analysis of certain structures are outside the scope of the current study.

## 4 Effects of Language Exposure on Sentence Comprehension

It is known that the effect of language exposure varies across linguistic domains and depending on clinical status (Blom and Paradis 2015; Elin Thordardottir 2011; Govindarajan and Paradis 2019; Smolander et al. 2021). The existing literature suggests that BiTD children with low L2 exposure levels might possess skills in receptive language that they are unable to demonstrate in productive language. Indeed, Chondrogianni and Marinis (2012) found differences in performance between TD and DLD children in comprehension, which were not detected in production due to insufficient exposure. They compared the performance of 6‐ to 9‐year‐old typically developing sequentially bilingual Turkish‐English‐speaking children to data from earlier studies on monolingual English‐speaking children with DLD. They found that BiTD children acquiring an L2 were sensitive to agrammaticality, despite variable production, whereas the monolingual DLD group was insensitive to these violations. In an earlier study, the same researchers found that comprehending tense morphology was not affected by input factors in BiTD children (Chondrogianni and Marinis 2011). Similarly, Unsworth (2014) proposed that there might be fewer input effects expected in the acquisition of underlying morphosyntactic rules and, hence, less bilingual effect in comprehension compared to production of language. She studied 7‐ to 9‐year‐old typically developing English‐speaking children who acquired Dutch as their second language and found that productive L2 verb morphology as well as vocabulary were predicted by the current amount of exposure, while comprehension of sentences with object scrambling was not (Unsworth 2016). Regarding object scrambling, Unsworth suggested that exposure might be irrelevant in explaining differences between bilingual children because the kind of information is unavailable in the input, referring to a ‘poverty of the stimulus’ problem. On the contrary, in a study by Chondrogianni and Marinis (2011), input effects were found in comprehending complex syntax, namely, passives and wh‐questions. These studies suggest that it depends on the exact phenomenon under investigation whether exposure effects can be found in comprehension.

Conflicting results can also be found in the studies using more holistic tests in assessing comprehension. De Cat and Melia (2022) studied English as a second language in TD children with several first language backgrounds and found no significant language exposure effects on L2 performance in the Sentence Structure subtest (SST) of CELF‐4, which can be considered analogous to the sentence comprehension test type used in the current study. The researchers suggested that the reason for exposure not being more important for the performance in SST was due to the fact that SST measures verbal reasoning skills rather than understanding complex structures. Contrary to the studies reporting fewer exposure effects on sentence comprehension, Elin Thordardottir et al. (2006) found a significant exposure effect in receptive syntax (Reynell Developmental Language Scales, RDLS) in typically developing simultaneously bilingual children and a significant difference in performance compared to monolingual peers. This difference may be related to age differences. Whereas the studies by Unsworth, for example, focused on school‐age children, Elin Thordardottir et al.’s (2006) study focused on young preschoolers. Further study of different age groups is, therefore, warranted.

To conclude, there is no consensus to date about the exposure effects on sentence‐level comprehension. The current literature indicates that exposure effects on sentence comprehension might vary depending on the test and item type, namely whether the test is able to reveal the skills in comprehending structural complexity or taps more importantly into, for example, inferencing skills or metalinguistic skills (as in grammatical judgement tasks) (De Cat and Melia 2022; Rusk et al. 2020; Unsworth 2016). Furthermore, the amount of information‐carrying words per sentence might be critical, since vocabulary and lexical retrieval are known to be very exposure dependent (Degani et al. 2019; Elin Thordardottir 2011; Frizelle et al. 2017).

There are no previous studies that would have compared exposure effects on sentence comprehension in BiTD and BiDLD. However, existing evidence looking at other language domains, for example, verb morphology, narratives and receptive and expressive vocabulary, suggests that depending on the domain or measure, BiTD and BiDLD groups can be differently affected by exposure (Blom and Paradis 2015; Govindarajan and Paradis 2019; Smolander et al. 2021). In many of the domains investigated, BiDLD children seem to have limitations in benefiting from exposure compared to their TD peers.

## 5 Classification Accuracy of Language Tests

It is very important to identify effective tests for distinguishing TD from language impairment in order to avoid problems of over‐ and under‐diagnosis. (Dollaghan 2007; Sansavini et al. 2021). One of the issues in evaluating diagnostic evidence is determining the accuracy of the test. This will not be determined by the examination of group mean differences alone (Ebert and Kohnert 2016; Elin Thordardottir et al. 2011). Test classification accuracy refers to productivity measures such as sensitivity, specificity and likelihood ratio (Dollaghan 2007). Sensitivity is the proportion of cases correctly classified as having the condition (DLD) (true positive). Specificity is the proportion of cases correctly classified as not having the condition (true negative). Likelihood ratios are preferably used in addition to sensitivity and specificity metrics because they are less affected by base rate (Dollaghan 2007). The positive likelihood ratio (LR+) reflects confidence that a positive (DLD) result is from a person with the condition rather than from a person without the condition. The negative likelihood ratio (LR−) reflects the confidence that a score in the non‐DLD range is from an unaffected person rather than from someone who has the condition. The above‐mentioned metrics are still often lacking regarding tests used in clinical work and research, or sensitivity and specificity do not always reach acceptable levels, with findings on different tests examined ranging as much as from 14% to 100% (Shahmahmood et al. 2016; Spaulding et al. 2006). Plante and Vance (1994) consider sensitivity and specificity above 90% to be good and above 80% to be fair. They recommend selecting tests with sensitivity and specificity of 0.80 and above, since the risk of misidentification increases significantly with lower rates of accuracy. In addition, an LR+ of 10 or more and an LR− of 0.10 or less are suggested to be informative in ruling DLD in or out (Deeks and Altman 2004).

Following these recommendations, the classification accuracy and diagnostic value of different assessment tools have been increasingly studied over the past few years. In particular, there has been an increase in evaluating the diagnostic accuracy of bilingual and L1 assessment tools (e.g., Peña et al. 2020). Studies assessing the applicability of L2 assessment tools have so far examined non‐word and sentence repetition (e.g., Armon‐Lotem and Meir 2016; Elin Thordardottir and Brandeker 2013; Paradis et al. 2013), productive morphosyntax (Gutiérrez‐Clellen and Simon‐Cereijido 2007), tense morphology (Paradis et al. 2013), narrative skills and vocabulary (Boerma and Blom 2017; Gillam et al. 2013; Paradis et al. 2013). For non‐word repetition, classification accuracy has mostly been found acceptable, whereas sentence repetition has in some studies shown lower specificity levels (Elin Thordardottir and Brandeker 2013). Vocabulary as a discriminator has proven to be less accurate in most studies. This can be explained by the fact that the most frequently used receptive and expressive vocabulary tests tap lexical retrieval, which in turn might be challenging even for bilingual TD children (Barak et al. 2022). This can be caused by competition between representations and weaker representations due to divided language usage. Sentence comprehension tasks have seldom been included. Gillam et al. (2013), however, investigated grammatical and narrative comprehension as part of their analysis. They found that, although sensitivity was high in grammatical comprehension, specificity suffered. Bonifacci et al. (2020) investigated many different language domains in their study and found a sensitivity of 0.85 and a specificity of 0.77 for morphosyntactic comprehension. More knowledge about the effects of language exposure on the diagnostic precision of the tests needs to be obtained.

## 6 The Current Study

We conducted a study of L2 sentence comprehension and exposure effects in sequentially bilingual children with TD and DLD (BiTD and BiDLD) children using two holistic offline sentence‐level comprehension tests. We predicted that assessing sentence comprehension can be a valuable domain in informing the differentiation of BiTD and BiDLD groups. In addition, we expected to find some but not a large effect of L2 exposure on sentence comprehension. This study adds to the literature by investigating L2 performance in the sparsely studied area of sentence comprehension, including in a BiDLD group in addition to the BiTD children. Furthermore, the study addresses the effect of exposure to L2 on the classification accuracy of the sentence comprehension tests. This study introduces data on children from several L1 backgrounds learning a non‐Indo‐European language, Finnish, as their L2. Introducing data on several L1 backgrounds when studying societal language performance is highly relevant for clinicians, as it depicts the reality of SLTs’ caseloads.

## 7 Methods

### 7.1 Participants

The participants in this study were a subgroup of a larger data set of the Helsinki longitudinal SLI study (HelSLI; see Laasonen et al. 2018). In the present study, 100 six‐year‐old (early) sequentially bilingual children participated, including 54 children with TD and 46 children with DLD (see Table 1 for the sample description).

**TABLE 1:** Descriptive statistics of age, age of onset, exposure and test scores (means, standard deviations, ranges, effect sizes and mean comparisons).

	Group						
	BiTD (*n* = 54)			BiDLD (*n* = 46)			
Variable	*M*	(*SD*)	Range (Max)	*M*	(*SD*)	Range (Max)	*d*
Age (months)	78.4	(3.3)	72–83	77.3	(3.5)	72–83	0.33
AoO (months)	27.9	(13.9)	1–70	30.6	(12.2)	9–57	−0.21
LoE (months)	50.6	(13.9)	12–78	46.7	(12.9)	17–70	0.29
L2Prop (%)	48	(10)	28–72	49	(8)	35–72	−0.14
CumLoE (months)	24.2	(8.4)	6.3–43.9	22.7	(7.1)	10.2–36.5	0.19
ExpLifetime (%)	31	(11)	8–57	30	(9)	14–51	0.16
PIQ	102.1	(13.5)	85–138	98.4	(12.1)	85–135	−0.11
Sent. Comp. Test RS	23.9	(2.7)	18–30 (30)	18.1	(4.4)	9–27 (30)	1.61^***^
Sent. Comp. Test StdS^a^	0.0	(1.0)	−2.2–2.3	−2.2	(1.6)	−5.6–1.2	
RDLS III, Comp., RS	55.7	(2.8)	47–62 (62)	49.7	5.3	36–60 (62)	1.45^***^
RDLS III, Comp., StdS^a^	0.0	(1.0)	−3.1–2.3	−2.1	(1.9)	−7.0–1.6	

*Note*: BiTD = Bilingual typically developing children; BiDLD = Bilingual children with developmental language disorder; *n* = Number of observations; M = Mean; SD = Standard deviation; d = Cohen's *d*, effect size; AoO (mo) = Age of onset L2 (months); LoE = Length of exposure in months; L2 Prop = Proportion of second language exposure; CumLoE = Cumulative length of exposure in months; ExpLifetime = Relative L2 exposure over lifetime; PIQ = Performance Intelligence Quotient; RS = Raw Score; StdS = Standard score; Sent. Comp. Test = Sentence Comprehension Test; RDLS III, Comp. = Reynell Developmental Language Scales III, Verbal comprehension scale.

***p < 0.001.

^a^ Standard scores are *z*‐scores based on the mean and standard deviation of the bilingual typically developing group in this data sample (*M* = 0, *SD* = 1).

Participant information regarding language environment was retrieved from the Finnish version of the Alberta Language Environment Questionnaire (ALEQ) (Paradis 2011; Smolander et al. 2013b). In our data, all children lived either with both of their parents or with their mother and another carer. Parents were the informants for the questionnaires, and parental informed consent was needed for participation in the study. Therefore, we will mainly use the word ‘parent’ in this study to refer to the carers of the child. Bilingual children in this sample were exposed to one language other than Finnish at home, spoken by both parents. Nevertheless, these L1 were numerous (27 different first languages in total, with the largest groups being Russian, Estonian, and Arabic, similarly in bilingual TD and DLD groups). This vast variety of first languages reflects the reality of SLT caseloads in the greater Helsinki area (see Bonifacci et al. 2020; De Cat and Melia 2022 for a similar design). Children in the study started acquiring Finnish as their L2 in day care, with various ages of onset for this sustained exposure to L2. Their attendance in the day care was continuous until the time of assessment. Most of the bilingual children (87% in both groups) were born in Finland. There was no statistically significant difference in this respect between the bilingual groups (*χ* ^2^(1) = 0.00, *p* = 0.990), indicating that children from the TD and DLD groups were equally often and mostly born in Finland. The mean age of onset (AoO) of sustained L2 exposure in both groups was approximately 2.5 years. Hence, the difference between the bilingual groups in the AoO of first exposure was not significant (see Table 1). Exposure to L2 over lifetime did not differ between the bilingual children with TD and DLD.

Of all BiTD children, 65% were boys, compared to 74% in the BiDLD group. The groups did not differ in their gender (*χ* ^2^(1) = 0.96, *p* = 0.327). Neither did they differ in age or nonverbal performance (Performance Intelligence Quotient, PIQ) (Table 1). In terms of maternal education (basic, secondary, and tertiary), the groups differed (*χ* ^2^(2) = 7.7, *p* < 0.05). This was mostly due to the higher proportion of higher education and the absence of a basic educational level in the BiTD group compared to the lower levels of education in the group of children with BiDLD.

Bilingual children with DLD were recruited when admitted for assessment to the Audiophoniatric Ward for children, Helsinki University Hospital, Finland, between 2013 and 2015. Participating in the study required parents’ informed consent. Ethical approval (approval reference number: § 248/2012) for the project was given by the ethical board of the Hospital District of Helsinki and Uusimaa.

Because of the lack of L1 tests for all the L1 languages of the children included in this study, as well as norm‐referenced L2 tests for bilingual children, the following inclusion criteria for children with DLD were used: Continuing and severe concern in language development (in both languages for bilinguals), language assessment carried out by the referring speech and language therapist (SLT), a report stating severe language difficulties, intervention or a follow‐up period in a communal setting, and finally referral to the Department of Phoniatrics, in which assessments for children with severe language difficulties are carried out. Developmental concerns regarding bilingual children's L1 were confirmed with a parent interview using a Finnish translation and adaptation of the Alberta Language and Development Questionnaire (ALDeQ) (Paradis et al. 2010; Smolander et al. 2013a) and other informal assessments of the L1 (e.g., language sampling, narrative production and comprehension, dynamic assessment, and observation). Both the interview and assessment were conducted with the help of an interpreter. Consistent and sustained Finnish day‐care exposure for at least 1 year was an inclusion criterion for bilingual children as well. Exclusion criteria were intellectual disability, diagnosed neurological impairment or disability, PIQ lower than 85, ASD, hearing impairment, or oral anomalies. All the children met the criteria for DLD according to the Finnish version of the International Statistical Classification of Diseases and Related Health Problems, ICD‐10 classification (THL 2011). All children with DLD had language difficulties, resulting in severe functional difficulties in spoken language comprehension and production that are unlikely to resolve without specialist help and have potentially significant consequences for educational and social outcomes (Bishop et al. 2017). The diagnostic decision was made by experts in secondary health care at a university clinic after collecting and reporting detailed case history information as well as information on language and communicative performance based on interviews, other informal assessments of the L1 and performance in formal SLT and neuropsychological assessments executed in the L2. In conclusion, instead of a gold standard that is largely based on certain cut‐off criteria in language testing and used for the identification of DLD in monolingual children, our inclusion criteria were based on the clinician's expertise, follow‐up, and continuing teacher and parent concern, which are supported as diagnostic measures in the literature (CATALISE, Bishop et al. 2017; Paradis et al. 2013).

Bilingual children with TD were recruited between 2014 and 2019 from daycares in the greater Helsinki area. Parents of children with TD were asked for consent, and children were recruited when the inclusion criteria were met. The inclusion criteria were no concerns about language development (in either of the languages for bilingual children), no history of language intervention other than SLT consultation for a minor articulation disorder and a regular day care exposure to the Finnish language for at least 1 year. A minor articulation disorder was considered a mild difficulty in producing a motorically challenging individual speech sound (for example, substitution of an apico‐alveolar tremulant with a uvular one). Children were recruited from the same city districts as the bilingual children with DLD, and we included a corresponding gender distribution, as in the DLD sample, to increase the comparability of the groups of interest. The exclusion criteria for children with BiTD were a PIQ lower than 85, language learning difficulties or other difficulties in development, and diagnosed difficulties in language and/or learning in parents or siblings.

### 7.2 Instruments and Data Collection

The instruments used in this study were a parent questionnaire for collecting information about language exposure and two offline sentence comprehension tests as dependent variables (see Laasonen et al. [2018] for the full test battery in the larger study, not presented here). A Finnish version of ALEQ (Paradis 2011; Smolander et al. 2013b) was used in this study as a basis for establishing the AoO of exposure for the L2 as well as cumulative language exposure (CumLoE) and consequently the proportionate exposure to the L2 over the child's lifetime (ExpLifetime). Information about AoO was elicited with the question ‘When did the child first start hearing Finnish on a regular basis?’ This regular exposure to L2 in day care surroundings was considered consistent and sustained when being continuous in a 5‐day‐a‐week part‐time care or three days in full‐time care. The exposure variable was established by taking the language exposure in months (LoE) from the start of L2 acquisition and weighing it with the proportion of L2 language exposure across the years during which L2 was acquired (see Table 1 and Table S1 for the exposure variables). This was then divided by chronological age in months to form the ExpLifetime variable. Questions considering three main categories were used to assess L2 exposure: L2 exposure in day care, language use at home with family members, and extracurricular and language activities at home or outside the home. Many of the questions were considered quantity‐based, but some of them combined quality aspects as well (see Paradis (2011), for richness of language). When composing the exposure variable and weighing its different aspects, the estimated maximum number of hours per week for the different interactions and activities comprising L1 and L2 was taken together. Hours allocated for siblings depended on the number of siblings, and mother‐child interaction received the rest of the exposure hours left after other activities (including the time spent with the other parent/carer). Proportionate exposure hours per language depended on the parents’ answers. A more detailed description of the exposure variables used in this study can be found in Smolander et al. (2021). However, in addition to the original exposure variable, the CumLoE exposure variable (cumulative language exposure in months) was further developed in this study to depict relative exposure over lifetime (ExpLifetime). One cumulative exposure variable was established, since the aim of this study was not to study the effects of different aspects or types of exposure. In addition, estimation of the maximum hours spent in different activities and weighing the length of exposure in months with the proportion of exposure for both languages based on the answers were used, since our experience is that parents have difficulties in answering these kinds of questionnaires retrospectively.

For this particular study, we used two standardised offline (aural) sentence comprehension tests as dependent variables, both of which were Finnish versions. The Sentence Comprehension Test (Lausetesti, Korpilahti 2001) was developed in Finnish for 5–6‐year‐old children. The child hears 30 sentences in total and selects a matching picture among the three pictures, including foils. The test is similar to other sentence‐level comprehension tests like the TROG‐2 (The Test for Reception of Grammar) and the CELF‐5 Sentence Comprehension subtest, except that the Finnish Sentence Comprehension Test includes three pictures to choose from instead of four. The test evaluates children's ability to process spoken sentences of increasing complexity and use adequate reasoning strategies. Sentences include different grammatical structures such as negation, past tense, passive voice, relative clauses, participial phrase construction, and morphological inflections such as singular versus plural and case inflection. A couple of the sentences include a reversible sequence of events, and only a few have abstract concepts such as comparative attributes. Hence, accumulated vocabulary knowledge is not at the centre of this test. The Sentence Comprehension Test manual itself does not provide reliability information. Hence, we executed a reliability analysis based on the larger data set used in the HelSLI study, including all the children older than 5 years old. We achieved acceptable internal consistency reliability (Cronbach's *α* = 0.80). The second test was the Reynell Developmental Language Scales III, verbal comprehension (Edwards et al. 1997), which has been adapted into Finnish as well as normed and standardised (Kortesmaa et al. 2001). The RDLS III Verbal Comprehension Scale requires comprehending sentences with increasing complexity and reasoning. Sentences presented require comprehending agents and actions, clausal constituents, thematic role assignment, complex grammar and inferencing. It includes both act‐out and multiple‐choice tasks, 62 items in total. The Finnish version of the test reports acceptable reliability (Cronbach's *α* = 0.80).

Since both sentence comprehension tests had a few missing values, we wanted to increase efficiency and reduce bias by imputing the missing values using the receptive vocabulary performance data collected from the same participants within the same period of time as part of the larger HelSLI study. These tests were the Finnish version ‘Suomenkielinen ymmärretyn sanaston testi’ of the Receptive One‐Word Picture Vocabulary Test, ROWPVT‐4 (Martin and Brownell 2010; Kunnari and Välimaa 2022) and the Finnish version of the Boehm Test of Basic Concepts, ‘Boehmin peruskäsitetesti’ (Boehm 1986; Heimo 1993).

The performance of bilingual children with TD and DLD in Finnish was assessed in the larger HelSLI study with a vast assessment battery, including both speech and language and neuropsychological tests. Assessments for children with DLD were carried out as a clinical routine for 3–5 consecutive days of the assessment period at the clinic. Children with TD were assessed during their day care in a quiet room. These assessments were performed by either a licensed SLT or a student under the supervision of an SLT or a neuropsychologist from the project. Nonverbal tests for establishing the PIQ were part of the psychological test battery. The language environment questionnaire (ALEQ) was conducted as a parent interview in the clinic for children with DLD and in day care for the TD group.

### 7.3 Data Analyses

Analyses predicting performance on the sentence comprehension tests were carried out separately for the two sentence comprehension tests using multiple regression in IBM SPSS Statistics (Version 29.0.2.0, 2023). Backward elimination of interaction terms was used to obtain a model that was as parsimonious as possible. Independent variables were mean‐centred to estimate unstandardised effects. However, centred variables were not used in the final model, which did not include interactions between the variables. Standardised variables were used to estimate the standardised effects and compare the performance between the TD and DLD groups in the two sentence comprehension tests (within‐between ANCOVA). The control variables in the analyses were age, three‐class maternal education according to the Finnish educational system (dummy coded, with basic level as the comparison group, having 0 in both dummy variables), and PIQ. The data were analysed cross‐sectionally, but assumptions of growth by exposure were made. We used two‐tailed statistical significance tests and set *α*  = 0.05.

Covariate‐specific receiver operating characteristic curve (ROC) analysis (Faraggi 2003) was used to estimate covariate‐specific thresholds for optimal sensitivity and specificity in the identification of DLD in the two sentence comprehension tests. Lee et al. (2023) suggest that presenting covariate‐specific ROC curves and the corresponding area under the curve (AUC) should be used in clinical decision‐making since diagnostic accuracy often varies depending on several factors rather than being a fixed property of a test. We included maternal education, PIQ, age and exposure as covariates. The cut‐off scores, as well as the sensitivity, specificity, AUC, likelihood ratios and confidence intervals, were calculated to determine classification accuracy (TD/DLD) for each sentence comprehension task. PIQ, age and exposure were included as continuous covariates. However, for the sake of clarity, we report the results for three exposure levels (0.20, 0.35, and 0.50), each for three age points (72, 78, and 84 months). These were the values in the current data that were considered the most informative since they were even numbers and reflected the distribution of the data, minimum, maximum, and midrange of exposures and age. The analyses were performed using the R 4.2.2 ROCnReg package (Rodríguez‐Álvarez and Inácio 2020).

A missing value analysis was performed for the dependent and control variables. There were a few missing values for the dependent variables (Sentence Comprehension Test *n* = 1 and RDLS III *n* = 4). They were imputed using the Expectation‐Maximisation algorithm (EM). We included age, gender, group (TD/DLD), PIQ, maternal education, both sentence comprehension test variables, and receptive vocabulary test variables from the larger HelSLI cross‐sectional data into the imputation model. The variables chosen for the imputation model were considered to predict the missing values and hence increase the precision and decrease the bias (Collins et al. 2001).

## 8 Results

### 8.1 Correlations Between Independent Variables and Their Relationship With Sentence Comprehension

Correlations between explanatory factors and sentence comprehension test results are presented in Table S1. The length of L2 exposure in months and the L2 proportion, which were used to establish further exposure variables, did not correlate (*r* = 0.01). For some children, the environment was more L1, and for some children, more L2 supportive, regardless of the LoE to L2. The two language measures studied here correlated with each other. PIQ had a low positive correlation with the Sentence Comprehension Test in the BiTD group. The correlation between these variables was not significant in the BiDLD group, and the PIQ‐RDLS III correlation was not detected in either of the groups. A low positive correlation between the Sentence Comprehension Test and exposure to L2 was detected in the BiTD group but not in the BiDLD group, whereas in the RDLS III, it was the opposite: L2 exposure correlated with the RDLS III in the BiDLD group (moderate positive correlation).

### 8.2 Models Predicting Sentence Comprehension

**FIGURE 1:**
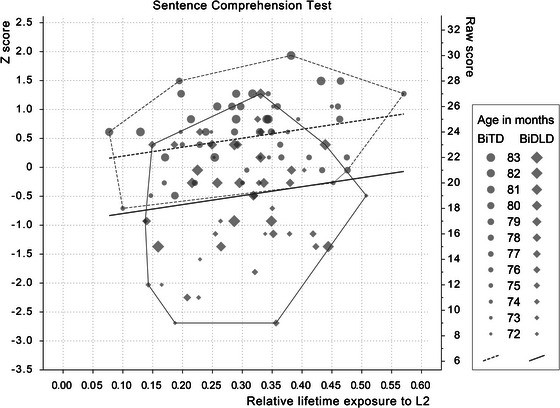
Sentence Comprehension Test score by relative L2 lifetime exposure. Regression lines from model are estimated with maternal education secondary level, age 78 months, PIQ 100. Convex hull around the groups. *Z*‐scores are based on the mean and SD of the sample (mean = 0, SD  = 1).

**FIGURE 2:**
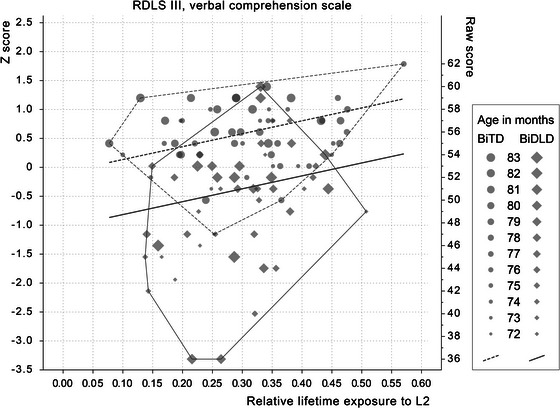
RDLS III Verbal Comprehension Scale score by relative L2 lifetime exposure. Regression lines from model are estimated with maternal education secondary level, age 78 months, PIQ 100. Convex hull around the groups. *Z*‐scores are based on the mean and SD of the sample (mean = 0, SD  = 1).

The role of control variables, age, gender, maternal education, and PIQ, was investigated. Age, maternal education, and PIQ were kept in the models for substantive reasons or because they differed between the groups. The effects of group (BiTD/BiDLD), exposure and AoO, as well as their interactions, were investigated to predict sentence comprehension. Backward elimination left only the main effects of exposure and control variables in the model. All effects are presented in Table 2, and the effects of exposure on sentence comprehension are shown in Figures 1 and 2.

**TABLE 2:** Results of the multiple regression analyses predicting sentence comprehension from group status (BiTD/BiDLD) and exposure.

	*B*	*β*	*p*
**Sent. Compr. Test (*R^2^_adj_* = 0.55)**			
MoEd2	1.64	0.18	0.042
MoEd3	2.29	0.24	0.005
Age (months)	0.46	0.35	< 0.001
PIQ	0.04	0.10	0.144
Group (BiTD/BiDLD)	−4.51	−0.50	< 0.001
ExpLifetime	7.03	0.16	0.026
**RDLS III, Comp. (*R^2^_adj_* = 0.48)**			
MoEd2	3.10	0.30	0.002
MoEd3	3.41	0.32	< 0.001
Age (months)	0.29	0.19	0.010
PIQ	−0.04	−0.10	0.185
Group (BiTD/BiDLD)	−4.85	−0.48	< 0.001
ExpLifetime	11.37	0.22	0.003

*Note*: Sent. Comp. Test = Sentence Comprehension Test, Lausetesti; RDLS III, Comp. = Reynell Developmental Language Scales III, Verbal Comprehension Scale; Group = Dummy variable, which before centring had 0 = Bilingual typically developing children and 1 = Bilingual children with DLD and after centring TD = −0.46 and DLD = 0.54, respectively; ExpLifetime = L2 relative exposure over lifetime; MoEd2 and MoEd3 = Dummy variables for maternal education (three levels in total), MoEd2 = secondary level = 1, MoEd3 = tertiary level = 1, reference category being lowest, basic level; PIQ = Performance Intelligence Quotient.

### 8.3 Performance and Predictors of the Sentence Comprehension Test

The model chosen for predicting performance in sentence comprehension was statistically significant for the Sentence Comprehension Test (*F*_6, 93_ = 21.1, *p* < 0.001, *R* ^2^_adj_ = 0.55). We found the main effects of group and exposure, which were the variables of greatest interest. These, alongside other effects, are presented in Table 2 and in Figure 1. The BiTD and BiDLD groups differed in their performance, with the BiTD group performing significantly better. Proportional lifetime exposure had a small effect (*β*_=_ 0.16). Even though all children were 6 years old (range 72–83 months in both groups), age had a main effect with a medium to large effect size. The BiTD and BiDLD groups both developed with increasing age within this narrow age range. Maternal education had a small to medium effect. Since there were no interactions between the main variables of interest, it can be concluded that the language exposure effects did not differ between the groups. Of all the significant predictors in the model, group (BiTD/BiDLD) was the strongest predictor of performance on the Sentence Comprehension Test (*β* = −0.50). Standardized regression coefficients indicated that in the group of BiTD children, a 10 percentage point (e.g., from 0.3 to 0.4) increase in the relative lifetime exposure resulted in less than half a point raw score increase in performance. For the BiDLD group, the increase was slightly greater (0.7 raw scores).

### 8.4 Performance and Predictors in the RDLS III Verbal Comprehension Scale

The chosen model explained the variation in the RDLS III Verbal Comprehension Scale well (*F*_6, 93_ = 16.5, *p* < 0.001, *R^2^_adj_* = 0.48). The effects on the RDLS III were similar to those on the Sentence Comprehension Test. The main effect of group was significant, indicating that the BiTD group performed significantly better compared to the peers with DLD (see Table 2). Exposure had a main effect with a small effect size (*β* = 0.22). The exposure effects did not differ between the groups. Maternal education and age had a main effect with a medium effect size. Again, group was the best predictor of performance. All effects are presented in Table 2 and in Figure 2. Standardised regression coefficients indicated a 0.6‐point increase in raw scores for the BiTD group when exposure increased by 10 percentage points, whereas there was a 1.2‐point raw score increase in the BiDLD group.

### 8.5 Comparing Standardised Performance Between Two Sentence Comprehension Tests

To find out whether one of the tests would be more informative in indicating group difference, we used within‐between ANCOVA. We focused on interactions of within‐factor (Sentence Comprehension Test–RDLS III) with explanatory variables (BiTD vs. BiDLD and exposure) while controlling for the same variables (age, PIQ, and maternal education) as in the multiple regression analyses. We did not find differing group or exposure effects on performance on the two sentence comprehension tests: Interactions of within‐factor × Group (*F* = 0.063, *p* = 0.803) and within‐factor × exposure (*F* = 0.764, *p* = 0.384) were non‐significant. In conclusion, this analysis did not show better differentiation for either of the two tests under any of the conditions.

### 8.6 Classification Accuracy of Sentence Comprehension Tests

**FIGURE 3:**
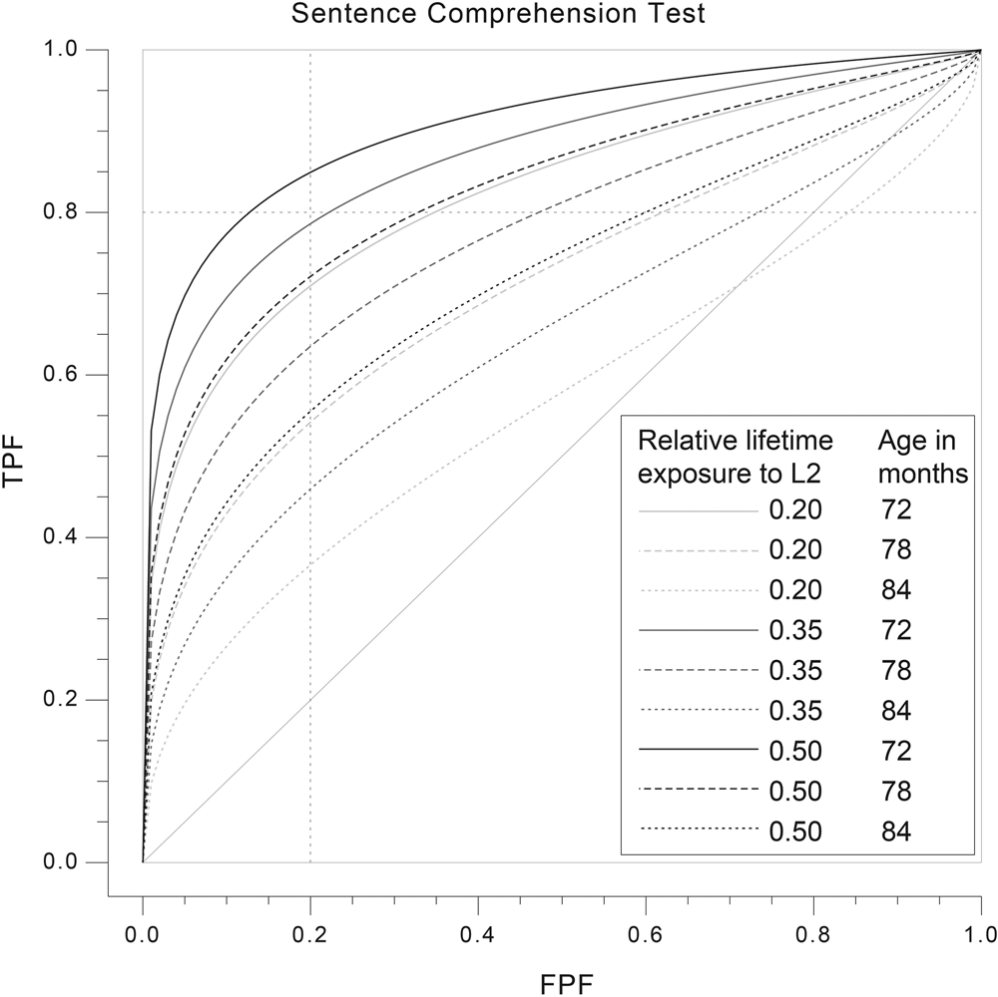
Estimated exposure‐specific ROC curves of Sentence Comprehension Test using the parametric frequentist approach of Faraggi (2003). Maternal education secondary level, PIQ 100. FPF, false positive fraction; TPF, true positive fraction.

**FIGURE 4:**
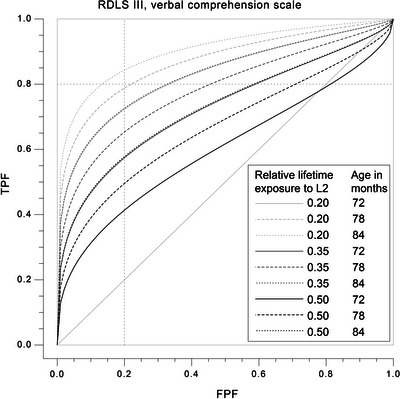
Estimated exposure‐specific ROC curves of RDLS III Verbal Comprehension Scale using the parametric frequentist approach of Faraggi (2003). Maternal education secondary level, PIQ 100. FPF, false positive fraction; TPF, true positive fraction.

We decided to use a sensitivity of 0.80 to set the optimal cut‐off score to secure, at a minimum, fair classification accuracy of those children affected by DLD, but also to secure reasonable specificity. The cut‐off scores, as well as the specificity, AUC, likelihood ratios and confidence intervals for three exposure levels (0.20, 0.35, and 0.50), each for three age points (72, 78, and 84 months), are reported in Table 3. In addition, covariate‐specific ROC curves are presented in Figures 3 and 4. Overall, the AUC values, as well as the sensitivity and specificity values of both tests, indicated that there was a great variation in classification accuracy depending on age and exposure. The specificity levels corresponding to a sensitivity of 0.80 for both tests were 0.87 at best, indicating fair classification accuracy. The corresponding positive likelihood ratio of both tests was 6.15, and the negative likelihood ratio was 0.23. From Table 3 and Figures 3 and 4, it can be observed that the two tests examined present opposite patterns in their classification accuracy when it comes to exposure effects. The Sentence Comprehension Test classified children better when the child had more exposure, whereas the RDLS III worked better when the child had lower levels of exposure to the L2. The Sentence Comprehension Test also showed less increase or decrease in classification accuracy at a given age when relative lifetime exposure changed compared to the RDLS III. In the Sentence Comprehension Test, a relative lifetime exposure of more than 40% resulted in an acceptable sensitivity and specificity of 0.80 when looking at the age at which classification accuracy was optimal. Fair sensitivity was also observed at lower levels of relative exposure to L2, but specificity suffered. For the RDLS III, specificity and sensitivity remained at an acceptable level (0.80 or higher) when relative lifetime exposure to L2 was 15%–25%. An increase in relative exposure resulted in a fairly rapid decrease in classification accuracy.

**TABLE 3:** Sensitivity and specificity estimates and likelihood ratios for sentence comprehension test scores based on covariate‐specific receiver operating curve analysis.

Test Relative L2 exposure	Optimal threshold	Sensitivity (TPF)	Specificity (1‐FPF)	AUC	95% CI (AUC)	LR+	LR−
**Sentence Comp. Test**							
**Age 72 months**							
ExpLifetime 0.20	19.4	0.80	0.65	0.82	0.59–0.96	2.29	0.31
ExpLifetime 0.35	19.8	0.80	0.78	0.87	0.69–0.98	3.64	0.26
ExpLifetime 0.50	20.1	0.80	0.87	0.91	0.70–0.99	6.15	0.23
**Age 78 months**							
ExpLifetime 0.20	23.0	0.80	0.38	0.71	0.47–0.89	1.29	0.53
ExpLifetime 0.35	23.3	0.80	0.53	0.77	0.61–0.92	1.70	0.38
ExpLifetime 0.50	23.7	0.80	0.67	0.83	0.60–0.97	2.42	0.30
**Age 84 months**							
ExpLifetime 0.20	26.5	0.80	0.16	0.57	0.24–0.85	0.95	1.25
ExpLifetime 0.35	26.9	0.80	0.27	0.64	0.37–0.89	1.10	0.74
ExpLifetime 0.50	27.2	0.80	0.40	0.72	0.38–0.94	1.33	0.50
**RDLS III, Verb. Comp**. **Age 72 months**							
ExpLifetime 0.20	52.1	0.80	0.67	0.83	0.60–0.97	2.42	0.61
ExpLifetime 0.35	54.8	0.80	0.41	0.73	0.45–0.92	1.36	0.34
ExpLifetime 0.50	57.5	0.80	0.19	0.60	0.23–0.91	0.99	1.05
**Age 78 months**							
ExpLifetime 0.20	53.1	0.80	0.78	0.87	0.72–0.97	3.64	0.26
ExpLifetime 0.35	55.8	0.80	0.54	0.78	0.62–0.92	1.74	0.37
ExpLifetime 0.50	58.5	0.80	0.29	0.67	0.37–0.90	1.13	0.69
**Age 84 months**							
ExpLifetime 0.20	54.2	0.80	0.87	0.90	0.71–0.99	6.15	0.23
ExpLifetime 0.35	56.9	0.80	0.67	0.83	0.61–0.96	2.42	0.30
ExpLifetime 0.50	59.6	0.80	0.42	0.73	0.37–0.95	1.38	0.48

*Note*: TPF = true positive fraction, FPF = false positive fraction; AUC = area under the curve; 95% CI = 95% bootstrapped confidence interval based on 1000 bootstrap samples; LR+ = positive likelihood ratio; LR− = negative likelihood ratio; Sentence Comp. Test = Sentence Comprehension Test (Lausetesti); RDLS III, Verb. Comp. = Reynell Developmental Language Scales III, Verbal Comprehension Scale; ExpLifetime = relative lifetime exposure to L2.

Other covariates had the following values: Maternal education = secondary level, and PIQ = 100.

## 9 Discussion

This study investigated the L2 sentence comprehension skills of BiTD and BiDLD children using two offline tests. We explored L2 exposure effects in both groups and studied the applicability of the sentence comprehension tests in distinguishing BiTD from BiDLD. To summarise the results, the performance of children with BiTD and BiDLD differed significantly in both sentence comprehension tests, with the BiTD group outperforming the BiDLD group. Relative lifetime exposure to L2 affected performance and did so to a similar degree in both groups, but with a small effect size. Concerning classification accuracy, however, the sensitivity and specificity of each of the two tests depended significantly on the relative lifetime exposure and age, even in this narrow age range.

### 9.1 Effects of DLD and Relative L2 Exposure on Sentence Comprehension

Even though only a few studies have investigated sentence comprehension, comparing BiTD and BiDLD groups (e.g., Girbau [2018]; see also Bonifacci et al. [2020] and Gillam et al. [2013] for classification accuracy in comprehension), it is not surprising that we found significant differences in performance between the two bilingual groups, given studies addressing monolingual children and basic mechanisms underlying DLD (Montgomery et al. 2021). The results of the current study are in line with previous research investigating L2 performance in bilingual children, which has shown lower performance across different language domains in children with BiDLD compared to BiTD children (e.g., Paradis et al. 2013; Smolander et al. 2021). Similar results in comprehension tasks were found by Girbau (2018), who related difficulties in children with DLD to challenges in storing representations on different linguistic levels in memory.

Considering the effects of exposure, the group‐level analysis revealed an effect of exposure as an explanatory factor of performance in sentence comprehension. The effect sizes were, however, smaller compared to the ones found in some other language domains, especially considering typically developing bilingual children (e.g., Blom and Paradis 2015; Elin Thordardottir 2011). It could be contemplated whether sentence comprehension in L2 might be substantially facilitated by cognitive maturity, some shared representations between the languages, inferential abilities and verbal reasoning skills—factors affecting sentence comprehension beyond accumulated linguistic skills in a particular language (e.g., Chondrogianni and Marinis 2012; De Cat and Melia 2022; Van Dijk et al. 2022). All these are factors that could allow the child to guess or infer the meaning, where language‐specific ability does not allow them to fully understand it.

Even though there was a difference in overall performance between the BiTD and BiDLD groups, they increased their performance to a similar degree as exposure increased. This reflects an opposite pattern to that of some previous studies targeting different domains. For example, Blom and Paradis (2015) and Govindarajan and Paradis (2019) found no effect of exposure on verb morphology performance and narrative skills in bilingual children with DLD, contrary to their TD peers. Similarly, in a study by Smolander et al. (2021), receptive vocabulary development was more resistant to exposure effects in the BiDLD group, whereas expressive vocabulary behaved similarly to the current study's exposure effects (similar cross‐sectional development in both groups). In the current study, it is encouraging that the development seen in the BiDLD group is not lagging even more behind the BiTD group, as seems to be the case in some other domains. This could be seen as evidence of bilingualism not exacerbating DLD (Degani et al. 2019; Paradis et al. 2003), but in the current study, comparisons to the performance of corresponding monolingual groups cannot be done.

It can be argued that the small exposure effect might be an artefact reflecting the properties of this offline test type, which can be criticised for not offering enough evidence about L2 children's comprehension of particular linguistic structures (De Cat and Melia 2022; Rusk et al. 2020). However, a valid and reliable test that includes a variety of structures chosen to depict the skills needed for successful overall comprehension in a classroom situation is of value. In addition, De Cat and Melia (2022) suggest that even though tests using the picture choice method to assess complex sentence comprehension do not necessarily measure the ability to comprehend complex, specific structures, they place heavy cognitive demands and might hence be an acceptable choice for identifying language disorders in bilingual children.

### 9.2 The Classification Accuracy of the Tests

In this study, the L2 sentence comprehension tests were acceptable discriminators of bilingual typical development and language disorder when looking at the optimal exposure level and age (AUC > 0.80, sensitivity and specificity 0.80–0.90). These sensitivity and specificity levels were comparable to those found, for example, in non‐word repetition (Armon‐Lotem and Meir 2016; Elin Thordardottir and Brandeker 2013) and somewhat higher than what Bonifacci et al. (2020) found for morphosyntactic comprehension using a measure very similar to the one in the current study. Gillam et al. (2013) instead found good sensitivity for grammatical understanding but unacceptable specificity of 0.69. The likelihood ratios of the current study at their best are suggesting greater differentiation ability in terms of establishing diagnosis than those of the study by Gillam and colleagues and the meta‐analysis by Dollaghan and Horner (2011) (LR+: 6.15 vs. 4.12–4.37). The ability to rule out DLD was, in turn, similar (LR−: 0.23 vs. 0.22–0.23). In the aforementioned studies, however, the results were from composite scores or pooled LR values across several language measures. Since the base rate of the sample in the current study is 50%, sensitivity, specificity, and likelihood ratio values should lead to the same conclusions about the accuracy of a given diagnostic measure (Dollaghan 2007). All in all, the results are likely to be rather suggestive in ruling in or out DLD rather than sufficient. Moreover, since the sensitivity and specificity were acceptable mainly within a narrow age and exposure range, it is obvious that the classification accuracy depended on exposure and that the tests investigated would not be able to correctly classify the whole age group with different exposure rates. This makes the exposure variable of practical relevance in sentence comprehension, as well as in other domains. Indeed, for example, Ebert and Kohnert (2016) suggest that including diagnostic accuracy measures can lead to different or even opposite conclusions from group‐level comparisons. Considering the fact that including exposure as a continuous variable in the ROC analysis was vital to interpreting the classification accuracy, it is clear that even though these data do not allow comparison to monolingual performance, using monolingual cut scores without knowing the effects of exposure on the performance on a certain test could lead to misdiagnosis of the bilingual children (see Elin Thordardottir 2015).

We are not aware of other studies that have used covariate‐specific ROC analysis in a similar manner as the current study, including exposure and age as continuous variables in the ROC analysis to study the classification accuracy of language tests. However, earlier studies on the effects of exposure on the classification accuracy of diagnostic or screening tests have suggested that language dominance has an effect and that the L2 learning phase is likely to affect the diagnostic value of the test (Altman et al. 2022; Gillam et al. 2013; Gutiérrez‐Clellen and Simon‐Cereijido 2007; Paradis et al. 2013). In the current study, the classification accuracy was the best in the Sentence Comprehension Test when there was at least 40% of relative L2 lifetime exposure, indicating more stable predictions at that point. These results can partly be related to the results of Gillam et al. (2013), who suggested that L2 testing could be used in diagnostics if children had been using the L2 at least 30% of the time for at least 1 year. Furthermore, Elin Thordardottir (2011) found that 40% relative lifetime exposure was necessary to score non‐significantly differently from monolingual children in receptive vocabulary in simultaneous bilinguals. A similar effect was shown for simultaneous and sequential school‐age children (Elin Thordardottir 2019). Based on this, these results indicate, not surprisingly, that a test developed for monolinguals applies most closely to those bilinguals whose ability in that language approaches that of monolinguals.

It is important to consider that the Sentence Comprehension Test and the RDLS III Verbal Comprehension Scale presented opposite patterns in terms of suitable exposure rates for acceptable classification accuracy. This was not shown in the analysis comparing the standardised performance on the two sentence comprehension tests. The RDLS III was a better test for classification in the case of less exposure compared to the Sentence Comprehension Test. This is an interesting result, since most of the previous studies suggest that the group differences and classification accuracy rather increase in the course of accumulating exposure (Altman et al. 2022; Paradis et al. 2013). The result is, however, in line with the fact that the verbal language scale of the RDLS III is considered a fairly easy test to accomplish for older children, hence losing its reliability in a monolingual context by the upper age range that it covers (Edwards et al. 1997; Kortesmaa et al. 2001). Consequently, it is losing its reliability for the bilingual children as they accumulate exposure. Conversely, the Sentence Comprehension Test was more accurate in classifying children with more exposure. The results of the current study also suggest that the Sentence Comprehension Test is more stable in classification accuracy across the exposure levels. These results reflect well the properties of the test, which was developed for older children and possesses norms for 5–6‐year‐old children, whereas the RDLS III is also used for younger children. These results point to the need to consider carefully how the age range targeted by a particular test relates to the population being tested. In this case, the two tests appear to be of considerably different overall difficulty levels, the RDLS III showing a substantial proportion of participants clustering at the high end in their performance, whereas for children with lower language ability and exposure levels, the test seemed to offer acceptable classification accuracy. Indeed, 32% of the participants scored 90% or higher on the RDLS III Verbal Comprehension Scale, and moreover, children with DLD had considerably high scores in concordance with higher rates of lifetime exposure to the L2. This consideration is also always pertinent for monolingual children, where it must be considered that the age range covered by a particular test is based on the norming for TD children, not that of children with DLD. These results point to the need to consider a test's difficulty level carefully and how it fits the language levels of monolingual and bilingual children differently.

### 9.3 Clinical Implications

Our results show that by using assessment tools developed for monolingual children, it is possible to find substantial differences between typical and disordered performance in bilingual children of various language backgrounds. This is highly important for the practical reason that bilingual children on the caseloads of SLTs are often from several different language backgrounds, the L2 being the mutual language of the child and the SLT (see, e.g., Bonifacci et al. 2020; De Cat and Melia 2022; Smolander et al. 2021). Based on our results, the applicability of the sentence‐level comprehension tests might be increased by the fact that exposure had only a small effect on performance. However, since the classification accuracy of the tests varied depending on exposure and age, it is recommended to have an exposure‐matched comparison group when interpreting the performance for diagnostic purposes. Additionally, opposite patterns in the classification accuracy as a function of exposure in the two tests call for profound knowledge of the test properties and individual consideration in choosing the method. Treatment planning can be further informed by item‐level analysis, considering individual challenges in morphosyntactic structures.

### 9.4 Limitations

We recognise some limitations in our study. Unfortunately, the typology of L1 as a background variable could not be taken into consideration because of the reduced sample size in different groups. Since the L1s were not assessed similarly to the L2 and there were no comparable tests for L1 assessments, conclusions about cross‐linguistic influence could not be made.

There is also the question of whether clinical offline tests, in general, can tease apart the reasons behind the performance. Both test types used in this study have been criticised for both under‐ and overestimating children's comprehension skills. However, based on the results and the prediction that poor performance could indicate subtle cognitive limitations typical in children with DLD (De Cat and Melia 2022), we consider these tests to be useful as an amendment to other assessment methods in bilingual children.

## 10 Conclusions

Language comprehension as a focus of interest is important because it is linked to other broader language outcomes and how children are able to communicate, participate, respond to intervention and learn in school. The results of the current study suggest that L2 sentence comprehension tests could be promising for informing the detection of language difficulties as an amendment to other assessment approaches in bilingual children with different L1 backgrounds. Even though, on group‐level comparisons, the effect of L2 exposure was small, it is highly recommended to have BiTD children as a reference for the BiDLD group and to consider exposure when comparing the groups for diagnostic and intervention planning purposes. The inclusion of the classification accuracy measure is suggested for interpreting the utility of an assessment tool, and covariate‐specific positivity thresholds are highly recommended to be used (Lee et al. 2023). Further clinical and experimental studies are needed in the field of sentence comprehension in sequentially bilingual children. Future research should include different explanatory factors (e.g., language typology, larger age range) and item‐level analysis in relation to clinical status (TD/DLD) and exposure level to add to the clinical utility and understanding of cross‐linguistic influence. In addition, since no one assessment tool or domain has been found to be sufficient for diagnosing DLD, the importance of sentence comprehension tests as classifiers should be considered as part of a larger assessment battery in future studies.

## Conflicts of Interest

The authors declare no conflicts of interest.

## Supporting information




**Supplemental Table**: Correlations between age, age of onset, exposure variables, main variables and control variables. Coefficients for the total sample are below the main diagonal and separate correlations for BiTD and BiDLD groups above it.

## Data Availability

The data that support the findings of this study are available on request from the corresponding author. The data are not publicly available due to privacy or ethical restrictions.

## References

[jlcd70125-bib-0001] Adlof, S. M. , and H. W. Catts . 2015. “Morphosyntax in Poor Comprehenders.” Reading and Writing 28: 1051–1070. 10.1007/s11145-015-9562-3. 27397969 PMC4934369

[jlcd70125-bib-0002] Altman, C. , E. Harel , N. Meir , P. Iluz‐Cohen , J. Walters , and S. Armon‐Lotem . 2022. “Using a Monolingual Screening Test for Assessing Bilingual Children.” Clinical Linguistics and Phonetics 36: 1132–1152. 10.1080/02699206.2021.2000644. 34844504

[jlcd70125-bib-0003] Arkkila, E. 2009. “Specific Language Impairment in Pre‐Adolescence, Adolescence, and Adulthood With Special Emphasis on Health‐Related Quality of Life.” PhD diss., University of Helsinki. http://urn.fi/URN:ISBN:978‐952‐10‐5808‐0.

[jlcd70125-bib-0083] Arkkila, E. , P. Räsänen , R. P. Roine , H. Sintonen , V. Saar , and E. Vilkman . 2009. “Health‐related quality of life of adolescents with childhood diagnosis of specific language impairment.” International Journal of Pediatric Otorhinolaryngology 73: 1288–1296. 10.1016/j.ijporl.2009.05.023. 19581006

[jlcd70125-bib-0004] Armon‐Lotem, S. , and N. Meir . 2016. “Diagnostic Accuracy of Repetition Tasks for the Identification of Specific Language Impairment (SLI) in Bilingual Children: Evidence From Russian and Hebrew.” International Journal of Language & Communication Disorders 51: 715–731. 10.1111/1460-6984.12242. 26990037

[jlcd70125-bib-0005] Barak, L. , T. Degani , and R. Novogdorsky . 2022. “Influences of Bilingualism and Developmental Language Disorder on How Children Learn and Process Words.” Developmental Psychology 58: 821–834. 10.1037/dev0001324. 35311315

[jlcd70125-bib-0006] Bishop, V. M. , M. J. Snowling , P. A. Thompson , and T. Greenhalgh , and the CATALISE consortium . 2017. “Phase 2 of CATALISE: A Multinational and Multidisciplinary Delphi Consensus Study of Problems With Language Development: Terminology.” Journal of Child Psychology and Psychiatry 58: 1–13. 10.1111/jcpp.12721. 28369935 PMC5638113

[jlcd70125-bib-0007] Blom, E. , and J. Paradis . 2015. “Sources of Individual Differences in the Acquisition of Tense Inflection in the Acquisition of Tense Inflection by English Second Language Learners With and Without Specific Language Impairment.” Applied Psycholinguistics 36: 953–976. 10.1017/S014271641300057X.

[jlcd70125-bib-0008] Boehm, A. E. 1986. Boehm Test of Basic Concepts. Psychological Corporation.

[jlcd70125-bib-0009] Boerma, T. , and E. Blom . 2017. “Assessment of Bilingual Children: What If Testing Both Languages Is Not Possible?” Journal of Communication Disorders 66: 65–76. 10.1016/j.jcomdis.2017.04.001. 28448800

[jlcd70125-bib-0010] Bonifacci, P. , E. Atti , M. Casamenti , B. Piani , M. Porrelli , and R. Mari . 2020. “Which Measures Better Discriminate Language Minority Bilingual Children With and Without Developmental Language Disorder? A Study Testing a Combined Protocol of First and Second Language Assessment.” Journal of Speech, Language, and Hearing Research 63: 1898–1915. 10.1044/2020_JSLHR-19-00100. 32516561

[jlcd70125-bib-0011] Chondrogianni, V. , and T. Marinis . 2011. “Differential Effects of Internal and External Factors on the Development of Vocabulary, Tense Morphology and Morpho‐syntax in Successive Bilingual Children.” Linguistic Approaches to Bilingualism 1: 318–345. 10.1075/lab.1.3.05cho.

[jlcd70125-bib-0012] Chondrogianni, V. , and T. Marinis . 2012. “Production and Processing Asymmetries in the Acquisition of Tense Morphology by Sequential Bilingual Children.” Bilingualism: Language and Cognition 15: 5–21. 10.1017/S1366728911000368.

[jlcd70125-bib-0013] Collins, L. M. , J. L. Schafer , and C.‐M. Kam . 2001. “A Comparison of Inclusive and Restrictive Strategies in Modern Missing Data Procedures.” Psychological Methods 6: 330–351. 10.1037/1082-989X.6.4.330. 11778676

[jlcd70125-bib-0014] Conti‐Ramsden, G. , K. Durkin , Z. Simkin , and E. Knox . 2009. “Specific Language Impairment and School Outcomes. I: Identifying and Explaining Variability at the End of Compulsory Education.” International Journal of Language & Communication Disorders 44: 15–35. 10.1080/13682820801921601. 18608604

[jlcd70125-bib-0015] Dasinger, L. 1997. “Issues in the Acquisition of Estonian, Finnish, and Hungarian: a Crosslinguistic Comparison.” In The Crosslinguistic Study of Language Acquisition, vol 4, edited by D. I. Slobin , 1–86. Lawrence Erlbaum Associates, Inc.

[jlcd70125-bib-0016] De Cat, C. , and T. Melia . 2022. “What Does the Sentence Structure Component of the CELF‐IV Index, in Monolinguals and Bilinguals?” Journal of Child Language 49: 423–450. 10.1017/S0305000920000823. 34229773

[jlcd70125-bib-0017] Deeks, J. J. , and D. G. Altman . 2004. “Diagnostic Tests 4: Likelihood Ratios.” British Medical Journal 329: 168–169. 10.1136/bmj.329.7458.168. 15258077 PMC478236

[jlcd70125-bib-0018] Degani, T. , V. Kreiser , and R. Novogrodsky . 2019. “The Joint Effects of Bilingualism, DLD and Item Frequency on Children's Lexical‐Retrieval Performance.” International Journal of Language & Communication Disorders 54: 485–498. 10.1111/1460-6984.12454. 30740851

[jlcd70125-bib-0019] Dollaghan, C. A. 2007. The Handbook of Evidence‐Based Practice in Communication Disorders. Paul H. Brookes Publishing Co.

[jlcd70125-bib-0020] Dollaghan, C. A. , and E. A. Horner . 2011. “Bilingual Language Assessment: A Meta‐Analysis of Diagnostic Accuracy.” Journal of Speech, Language, and Hearing Research 54: 1077–1088. 10.1044/1092-4388(2010/10-0093). 21106696

[jlcd70125-bib-0021] Ebert, K. D. , and K. Kohnert . 2016. “Language Learning Impairment in Sequential Bilingual Children.” Language Teaching 49: 301–338. 10.1017/S0261444816000070.

[jlcd70125-bib-0022] Ebert, K. D. , and M. Reilly . 2022. “Predictors of Language Proficiency in School‐Age Spanish‐English Bilingual Children With and Without Developmental Language Disorder.” Bilingualism 25: 296–306. 10.1017/S1366728921000985. 36051378 PMC9432479

[jlcd70125-bib-0023] Edwards, S. , P. Fletcher , M. Garman , A. Hughes , C. Letts , and I. Sinka . 1997. Reynell Developmental Language Scales III. NFER Nelson. Translation and standardisation of the Finnish version by M. Kortesmaa , K. Heimonen , H. Merikoski , M.‐L. Warma , and V. Varpela , for Psykologien Kustannus Oy, 2001.

[jlcd70125-bib-0024] Faraggi, D. 2003. “Adjusting Receiver Operating Characteristic Curves and Related Indices for Covariates.” *Journal of the Royal Statistical Society. Series D* 52: 179–192. 10.1111/1467-9884.00350.

[jlcd70125-bib-0025] Frizelle, P. , J. Harte , K. O'Sullivan , P. Fletcher , and F. Gibbon . 2017. “The Relationship Between Information Carrying Words, Memory and Language Skills in School Age Children With Specific Language Impairment.” PLoS ONE 12, no. 7: e0180496. 10.1371/journal.pone.0180496. 28672043 PMC5495434

[jlcd70125-bib-0026] Gillam, R. B. , E. D. Peña , L. M. Bedore , T. M. Bohman , and A. Mendez‐Perez . 2013. “Identification of Specific Language Impairment in Bilingual Children: I. Assessment in English.” Journal of Speech, Language, and Hearing Research 56: 1813–1823. 10.1044/1092-4388(2013/12-0056). PMC5902172 23882008

[jlcd70125-bib-0027] Girbau, D. 2018. “Direct Object Pronoun Sentence Processing in Spanish‐English Children With/Without Specific Language Impairment and Adults: A Cross‐Modal Priming Study.” Journal of Communication Disorders 72: 97–110. 10.1016/j.jcomdis.2018.01.003. 29426787

[jlcd70125-bib-0028] Govindarajan, K. , and J. Paradis . 2019. “Narrative Abilities of Bilingual Children With and Without Developmental Language Disorder (SLI): Differentiation and the Role of Age and Input Factors.” Journal of Communication Disorders 77: 1–16. 10.1016/j.jcomdis.2018.10.001. 30408604

[jlcd70125-bib-0029] Gutiérrez‐Clellen, V. F. , and G. Simon‐Cereijido . 2007. “The Discriminant Accuracy of a Grammatical Measure With Latino English‐Speaking Children.” Journal of Speech, Language, and Hearing Research 50: 968–981. 10.1044/1092-4388(2007/068). PMC3367477 17675599

[jlcd70125-bib-0085] Heimo, H. 1993. Boehmin peruskäsitetesti. Psykologien Kustannus Oy.

[jlcd70125-bib-0030] Hsu, H. J. , B. Tomblin , and M. H. Christiansen . 2014. “Impaired Statistical Learning of Non‐Adjacent Dependencies in Adolescents With Specific Language Impairment.” Frontiers in Psychology 175: 1–10. 10.3389/fpsyg.2014.00175. PMC3944677 24639661

[jlcd70125-bib-0031] Huumo, T. 2023. Introduction. A Cognitive Linguistic Account of the Finnish Cases. In The Finnish Case System. Cognitive Linguistic Perspectives, edited by M. Jaakola and T. Onikki‐Rantajääskö , 10–37. Finnish Literary Society. 10.21435/sflin.23.

[jlcd70125-bib-0032] Hyönä, J. , and H. Hujanen . 1997. “Effects of Case Marking and Word Order on Sentence Parsing in Finnish: An Eye Fixation Analysis.” Quarterly Journal of Experimental Psychology 50: 841–858. 10.1080/713755738.

[jlcd70125-bib-0033] IBM Corp . 2023. IBM SPSS Statistics for Windows (Version 29.0.2.0). [Computer software]. IBM Corp.

[jlcd70125-bib-0036] Jones, S. D. , and G. Westermann . 2021. “Predictive Processing and Developmental Language Disorder.” Journal of Speech, Language, and Hearing Research 64: 181–185. 10.1044/2020_JSLHR-20-00409 33375825

[jlcd70125-bib-0037] Karlsson, F. 2015. Finnish. An Essential Grammar. Routledge.

[jlcd70125-bib-0038] Kittilä, S. , J. Laakso , and J. Ylikoski . 2022. “Case.” In The Oxford Guide to the Uralic Languages, edited by M. Bakró‐Nagy , J. Laakso , and E. Skribnik , 879–893. Oxford University Press. 10.1093/oso/9780198767664.003.0044.

[jlcd70125-bib-0039] Korpilahti, P. 2001. Lausetesti *(Sentence Comprehension Test)* . Language & Communication Care.

[jlcd70125-bib-0040] Kortesmaa, M. , K. Heimonen , H. Merikoski , M.‐L. Warma , and V. Varpela . 2001. Reynellin kielellisen kehityksen testi. Reynell Developmental Language Scales III. Psykologien kustannus Oy.

[jlcd70125-bib-0041] Kunnari, S. , L. Nieminen , and P. Torvelainen . 2016. “FIN‐LARSP: Morphosyntactic Profiling of Finnish Children.” In Profiling Grammar: More Languages of LARSP, edited by P. Fletcher , M. Ball , and D. Crystal , 64–79. Multilingual Matters. 10.21832/9781783094875-006.

[jlcd70125-bib-0042] Kunnari, S. , T. Savinainen‐Makkonen , L. Leonard , et al. 2011. “Children With Specific Language Impairment in Finnish: The Use of Tense and Agreement Inflections.” Journal of Child Language 38: 999–1027. 10.1017/S0305000910000528. 21281548 PMC3600168

[jlcd70125-bib-0084] Kunnari, S. , and T. Välimaa . 2022. Suomenkielinen ymmärretyn sanaston testi. Puheterapeuttien Kustannus Oy.

[jlcd70125-bib-0043] Laasonen, M. , S. Smolander , P. Lahti‐Nuuttila , et al. 2018. “Understanding Developmental Language Disorder—The Helsinki Longitudinal SLI Study (HelSLI): A Study Protocol.” BMC Psychology 6: 24. 10.1186/s40359-018-0222-7. 29784061 PMC5963016

[jlcd70125-bib-0044] Lee, J. , N. van Es , T. Takada , the IPD study team , et al. 2023. “Covariate‐Specific ROC Curve Analysis Can Accommodate Differences Between Covariate Subgroups in the Evaluation of Diagnostic Accuracy.” Journal of Clinical Epidemiology 160: 14–23. 10.1016/j.jclinepi.2023.06.001. 37295733

[jlcd70125-bib-0045] Leonard, L. B. 2014. Children With Specific Language Impairment. The MIT Press. 10.7551/mitpress/9152.001.0001.

[jlcd70125-bib-0046] Leonard, L. B. 2022. “Developmental Language Disorder and the Role of Language Typology.” Enfance 1: 25–39. 10.3917/enf2.221.0025.

[jlcd70125-bib-0047] Leonard, L. B. , S. Kunnari , T. Savinainen‐Makkonen , et al. 2014. “Noun Case Suffix Use by Children With Specific Language Impairment: An Examination of Finnish.” Applied Psycholinguistics 35: 833–854. 10.1017/S0142716412000598. 25995529 PMC4435715

[jlcd70125-bib-0048] MacWhinney, B. 2005. “Extending the Competition Model.” International Journal of Bilingualism 9: 69–84. 10.1177/13670069050090010501.

[jlcd70125-bib-0049] MacWhinney, B. , and C. Pléh . 1988. “The Processing of Restrictive Relative Clauses in Hungarian.” Cognition 29: 95–141. 10.1016/0010-0277(88)90034-0. 3168422

[jlcd70125-bib-0050] Martin, N. , and R. Brownell . 2010. Receptive One‐Word Picture Vocabulary Test 4. Academic Therapy Publications.

[jlcd70125-bib-0051] Montgomery, J. W. , R. B. Gillam , and J. L. Evans . 2021. “A New Memory Perspective on the Sentence Comprehension Deficits of School‐Age Children With Developmental Language Disorder: Implications for Theory, Assessment, and Intervention.” Language, Speech, and Hearing Services in Schools 52: 449–466. 10.1044/2021_LSHSS-20-00128. 33826402 PMC8711711

[jlcd70125-bib-0052] Paradis, J. 2011. “Individual Differences in Child English Second Language Acquisition. Comparing Child‐Internal and Child‐External Factors.” Linguistic Approaches to Bilingualism 1: 213–237. 10.1075/lab.1.3.01par.

[jlcd70125-bib-0053] Paradis, J. , M. Crago , F. Genesee , and M. Rice . 2003. “French‐English Bilingual Children With SLI: How Do They Compare With Their Monolingual Peers?” Journal of Speech, Language, and Hearing Research 46: 113–127. 10.1044/1092-4388(2003/009). 12647892

[jlcd70125-bib-0054] Paradis, J. , K. Emmerzael , and T. S. Duncan . 2010. “Assessment of English Language Learners: Using Parent Report on First Language Development.” Journal of Communication Disorders 43: 474–497. 10.1016/j.jcomdis.2010.01.002. 20304411

[jlcd70125-bib-0055] Paradis, J. , P. Schneider , and T. Sorenson Duncan . 2013. “Discriminating Children With Language Impairment Among English‐Language Learners From Diverse First‐Language Backgrounds.” Journal of Speech, Language, and Hearing Research 56: 971–981. 10.1044/1092-4388(2012/12-0050). 23275391

[jlcd70125-bib-0056] Peña, E. D. , L. M. Bedore , M. J. Lugo‐Neris , and N. Albudoor . 2020. “Identifying Developmental Language Disorder in School Age Bilinguals: Semantics, Grammar, and Narratives.” Language Assessment Quarterly 17: 541–558. 10.1080/15434303.2020.1827258. 35895289 PMC9311479

[jlcd70125-bib-0057] Plante, E. , and R. Vance . 1994. “Selection of Preschool Language Tests: A Data‐Based Approach.” Language, Speech and Hearing Services in School 25: 15–24.

[jlcd70125-bib-0058] Reilly, S. , F. Cook , E. L. Bavin , et al. 2018. “Cohort Profile: the Early Language in Victoria Study (ELVS).” International Journal of Epidemiology 47: 11–20. 10.1093/ije/dyx079. 29040559

[jlcd70125-bib-0059] Reyes, I. , and A. E. Hernández . 2006. “Sentence Interpretation Strategies in Emergent Bilingual Children and Adults.” Bilingualism: Language and Cognition 9: 51–69. 10.1017/S1366728905002373.

[jlcd70125-bib-0060] Rice, M. L. , K. Wexler , and S. M. Redmond . 1999. “Grammaticality Judgements of an Extended Optional Infinitive Grammar: Evidence From English‐Speaking Children With Specific Language Impairment.” Journal of Speech, Language, and Hearing Research 42: 943–961. 10.1044/jslhr.4204.943. 10450913

[jlcd70125-bib-0034] Rodríguez‐Álvarez, M. X. , and V. Inácio . 2020. “ROCnReg: An R Package for Receiver Operating Characteristic Curve Inference With and Without Covariate Information.” arXiv:2003.13111 [stat.ME]. October 30. 10.32614/rj-2021-066.

[jlcd70125-bib-0061] Rusk, B. V. , J. Paradis , and J. Järvikivi . 2020. “Comprehension of English Plural‐Singular Marking by Mandarin‐L1, Early L2‐Immersion Learners.” Applied Psycholinguistics 41: 547–577. 10.1017/S0142716420000089.

[jlcd70125-bib-0062] Sansavini, A. , M. E. Favilla , M. T. Guasti , et al. 2021. “Developmental Language Disorder: Early Predictors, Age for the Diagnosis, and Diagnostic Tools. A Scoping Review.” Brain Sciences 11: 1–38. 10.3390/brainsci11050654. PMC8156743 34067874

[jlcd70125-bib-0063] Shahmahmood, T. M. , S. Jalaie , Z. Soleymani , F. Haresabadi , and P. Nemati . 2016. “A Systematic Review on Diagnostic Procedures for Specific Language Impairment: The Sensitivity and Specificity Issues.” Journal of Research in Medical Sciences 21: 1–16. 10.4103/1735-1995.189648. 27904612 PMC5122002

[jlcd70125-bib-0064] Slobin, D. I. , and T. G. Bever . 1982. “Children Use Canonical Sentence Schemas: A Crosslinguistic Study of Word Order and Inflections.” Cognition 12: 229–265. 10.1016/0010-0277(82)90033-6. 6891309

[jlcd70125-bib-0065] Smolander, S. , M. Laasonen , E. Arkkila , P. Lahti‐Nuuttila , and S. Kunnari . 2021. “L2 Vocabulary Acquisition of Early Sequentially Bilingual Children With TD and DLD Affected Differently by Exposure and Age of Onset.” Journal of Language and Communication Disorders 56: 72–89. 10.1111/1460-6984.12583. 33179849

[jlcd70125-bib-0066] Smolander, S. , M. Laasonen , S. Kunnari , and E. Service . 2013a. “Finnish Version of The Alberta Language Development Questionnaire (ALDeQ).” Published June 2013. https://www.hus.fi/tutkimus‐ja‐opetus/tutkimukset/helsli‐seurantatutkimus#tuloksia.

[jlcd70125-bib-0067] Smolander, S. , M. Laasonen , S. Kunnari , and E. Service . 2013b. “Finnish Version of The Alberta Language Environment Questionnaire (ALEQ).” Published June 2013. https://www.hus.fi/tutkimus‐ja‐opetus/tutkimukset/helsli‐seurantatutkimus#tuloksia.

[jlcd70125-bib-0068] Spaulding, T. J. , E. Plante , and K. A. Farinella . 2006. “Eligibility Criteria for Language Impairment: Is the Low End of Normal Always Appropriate?” Language, Speech, and Hearing Services in Schools 37: 61–72. 16615750 10.1044/0161-1461(2006/007)

[jlcd70125-bib-0069] THL . 2011. “Third edition of the Finnish version of the International Statistical Classification of Diseases and Related Health Problems.” https://urn.fi/URN:NBN:fi‐fe201205085423.

[jlcd70125-bib-0070] Thordardottir, E. 2011. “The Relationship Between Bilingual Exposure and Vocabulary Development.” International Journal of Bilingualism 15: 426–445. 10.1177/1367006911403202. 25029077

[jlcd70125-bib-0071] Thordardottir, E. 2015. “Proposed Diagnostic Procedures for Use in Bilingual and Cross‐Linguistic contexts.” In Assessing Multilingual Children: Disentangling Bilingualism From Language Impairment, edited by S. Armon‐Lotem , J. de Jong , and N. Meir , 331–358. Multilingual Matters. 10.21832/9781783093137-014.

[jlcd70125-bib-0072] Thordardottir, E. 2016. “Grammatical Morphology Is Not a Sensitive Marker of Language Impairment in Icelandic in Children Aged 4–14 Years.” Journal of Communication Disorders 62: 82–100. 10.1016/j.jcomdis.2016.06.001. 27314205

[jlcd70125-bib-0073] Thordardottir, E. 2019. “Amount Trumps Timing in Bilingual Vocabulary Acquisition: Effects of Input in Simultaneous and Sequential School‐Age Bilinguals.” International Journal of Bilingualism 23: 236–255. 10.1177/1367006917722418.

[jlcd70125-bib-0074] Thordardottir, E. , and M. Brandeker . 2013. “The Effect of Bilingual Exposure Versus Language Impairment on Nonword Repetition and Sentence Imitation Scores.” Journal of Communication Disorders 46: 1–16. 10.1016/j.jcomdis.2012.08.002. 23021785

[jlcd70125-bib-0075] Thordardottir, E. , E. Kehayia , B. Mazer , et al. 2011. “Sensitivity and Specificity of French Language and Processing Measures for the Identification of Primary Language Impairment at Age 5.” Journal of Speech, Language, and Hearing Research 54: 580–597. 10.1044/1092-4388(2010/09-0196). 21081674

[jlcd70125-bib-0076] Thordardottir, E. , A. Rothenberg , M. Rivard , and R. Naves . 2006. “Bilingual Assessment: Can Overall Proficiency be Estimated From Separate Measurement of Two Languages?” Journal of Multilingual Communication Disorders 4: 1–21. 10.1080/14769670500215647.

[jlcd70125-bib-0077] Tomblin, J. B. , N. L. Records , P. Buckwalter , X. Zhang , E. Smith , and M. O'Brien . 1997. “Prevalence of Specific Language Impairment in Kindergarten Children.” Journal of Speech, Language, and Hearing Research 40: 1245–1260. 10.1044/jslhr.4006.1245. PMC5075245 9430746

[jlcd70125-bib-0078] Unsworth, S. 2014. “Comparing the Role of Input in Bilingual Acquisition Across Domains.” In Input and Experience in Bilingual Development, edited by T. Grüter and J. Paradis , 181–201. John Benjamins Publishing Company. 10.1075/tilar.13.10uns.

[jlcd70125-bib-0079] Unsworth, S. 2016. “Early Child L2 Acquisition: Age or Input Effects? Neither or Both?” Journal of Child Language 43: 608–634. 10.1017/S030500091500080X. 26915919

[jlcd70125-bib-0080] Vainio, S. , A. Pajunen , and J. Hyönä . 2014. “L1 and L2 Word Recognition in Finnish—Examining L1 Effects on L2 Processing of Morphological Complexity and Morphophonological Transparency.” Studies in Second Language Acquisition 36: 133–162. 10.1017/S0272263113000478.

[jlcd70125-bib-0081] Van der Lely, H. K. J. , and S. Pinker . 2014. “The Biological Basis of Language: Insight From Developmental Grammatical Impairments.” Trends in Cognitive Science 18: 1–10. 10.1016/j.tics.2014.07.001. 25172525

[jlcd70125-bib-0082] Van Dijk, C. , T. Dijkstra , and S. Unsworth . 2022. “Cross‐Linguistic Influence During Online Sentence Processing in Bilingual Children.” Bilingualism: Language and Cognition 25: 691–704. 10.1017/S1366728922000050.

