# Supplementary material for: Sentence Comprehension and L2 Exposure Effects in 6‐Year‐Old Sequentially Bilingual Children With Typical Development and Developmental Language Disorder
Source: Int J Lang Commun Disord. 2025 Sep 28;60(6):e70125. doi: 10.1111/1460-6984.70125 (PMC12477347; doi:10.1111/1460-6984.70125)
Supplement: Supplementary file 1 — Supplemental Table: Correlations between age, age of onset, exposure variables, main variables and control variables. Coefficients for the total sample are below the main diagonal and separate correlations for BiTD and BiDLD groups above it. [file JLCD-60-0-s001.docx]

| BiTD  BiDLD | | | | | | | | | |
| --- | --- | --- | --- | --- | --- | --- | --- | --- | --- |
| **Variable** | **1.** | **2.** | **3.** | **4.** | **5.** | **6.** | **7.** | **8.** | **9.** |
| 1. Age |  | .13 | .11 | -.09 | .02 | -.10 | .04 | .40** | .38** |
|  |  | -.07 | .33* | -.18 | .20 | .06 | -.16 | .42** | .12 |
| 2. AoO | .03 |  | -.97*** | -.09 | -.81*** | -.82*** | -.26 | -.25 | -.19 |
|  |  |  | -.96*** | -.01 | -.83*** | -.83*** | -.17 | -.25 | -.34* |
| 3. LoE | .23* | -.97*** |  | .07 | .82*** | .80*** | .27 | .35^†^ | .28 |
|  |  |  |  | -.06 | .84*** | .81*** | .12 | .35* | .35* |
| 4. L2 Prop | -.14 | -.05 | .01 |  | .61*** | .63*** | -.18 | .17 | .04 |
|  |  |  |  |  | .46^††^ | .50*** | .04 | .11 | .17 |
| 5. CumLoE | .11 | -.82*** | .83*** | .55*** |  | .99*** | .09 | .34* | .26 |
|  |  |  |  |  |  | .99*** | .10 | .23 | .39** |
| 6. ExpLifetime | -.02 | -.83*** | .80*** | .55*** | .99*** |  | .09 | .30* | .22 |
|  |  |  |  |  |  |  | .12 | .18 | .38** |
| 7.PIQ | -.02 | -23.* | .22* | -.10 | .11 | .11 |  | .31* | .02 |
|  |  |  |  |  |  |  |  | .02 | -17 |
| 8. Sent. Comp. Test | .41*** | -.25* | .35*** | -.03 | .27** | .22* | .20* |  | .36** |
|  |  |  |  |  |  |  |  |  | .55*** |
| 9. RDLS III, Comp. | .26** | -.27** | .33*** | .04 | .31** | .28** | .01 | .69*** |  |

Supplemental Table. Correlations between age, age of onset, exposure variables, main variables and control variables. Coefficients for the total sample are below the main diagonal and separate correlations for BiTD and BiDLD groups above it.

Note. BiTD = Bilingual typically developing children; BiDLD = Bilingual children with developmental language disorder; AoO = Age of onset; LoE = Language exposure in months; L2 Prop = Relative exposure to L2; CumLoE = Cumulative language exposure; ExpLifetime = Relative L2 exposure over lifetime; PIQ = Perfomance Intelligence Quotient; Sent. Comp. Test = Sentence Comprehension Test; RDLS III, Comp. = Reynell Developmental Language Scales III, Verbal comprehension scale.

* *p* < .05, ** *p* < .01, *** *p* < .001, ^†^ *p* = .0102, ^††^ *p* = .0012
